# Transient Perivascular Inflammation of the Carotid Artery as a Poorly Recognized Cause of Neck Pain

**DOI:** 10.1055/a-2223-5580

**Published:** 2024-02-07

**Authors:** Sophie Greutert, Tatiana Schlomer, Marc Righini

**Affiliations:** 1 Division of Angiology and Hemostasis, Geneva University Hospitals and Faculty of Medicine, Geneva, Switzerland

**Keywords:** TIPIC syndrome, temporary perivascular infiltration, carotidynia, Fay syndrome, neck pain

## Abstract

Transient perivascular inflammation of the carotid artery (TIPIC) syndrome, historically named idiopathic carotidynia or Fay syndrome, is a rare condition characterized by inflammation and pain in the carotid artery. The diagnosis requires a specific clinical–radiological presentation. We describe a 37-year-old female who presented with headaches and left neck pain and was diagnosed with TIPIC syndrome with temporary perivascular infiltration.

## Introduction

Transient perivascular inflammation of the carotid artery (TIPIC) syndrome, historically named idiopathic carotidynia, is a rare condition characterized by inflammation and pain in the carotid artery. It was first described as carotidynia in 1927. 1 It was classified as an idiopathic neck pain syndrome by the International Classification of Headache Disorders (ICHD) in 1988. 2 At that time, diagnosis of carotidynia or Fay syndrome could be evoked in the following situations: pain over the affected side of the neck, associated with tenderness, swelling, or increased pulsation on local pressure application and the concomitant exclusion of structural causes of carotid pain or other local causes with a self-resolution in less than 14 days. 2 However, due to the lack of symptom's specificity and after several discussions and arguments, 3 4 ICHD decided to remove carotidynia as a distinct entity. 5

Since 1927, few cases presenting unilateral or bilateral tenderness or pain located in the carotid bifurcation area associated with atypical ultrasound imaging presentation have been reported. In 2017, Lecler et al have enlightened the description on an unclassified, clinical–radiological entity that has been proposed to be described under the acronym TIPIC syndrome. 6 Our case is relevant as it is a poorly recognized cause of neck pain. Our goal is to reinforce the necessity of better knowledge regarding the diagnosis and follow-up of this rare clinical entity.

## Case Presentation

We are reporting the case of a 37-year-old female who consulted her general practitioner for increasing left neck pain, triggered by head movement or palpation, without a history of trauma. In addition, she also reported fatigue without fever or any other neurological symptoms. Her history was notable for class I obesity and migraines.

First examination showed a normal clinical status, especially no face or neck swelling nor lymphadenopathy, whereas a tenderness at palpation and worsening by head movement was noticed at the level of the left carotid bifurcation.

**Fig. 1:**
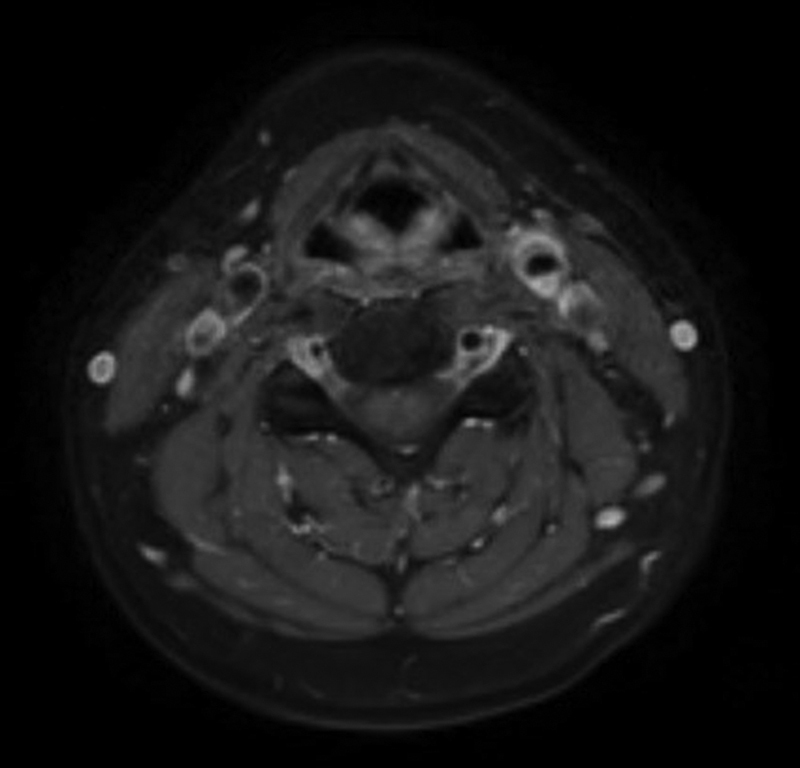
Magnetic resonance imaging at diagnosis.

Ultrasound imaging described an increased mural thickening measured up to 1.8 mm associated with hyperechogenicity, corresponding to eccentric perivascular infiltration (PVI) at that level, without lumen narrowing or hemodynamic change in Doppler mode. No dissection was diagnosed and the intima-media thickness was normal. Radiological status was completed with a contrast-enhanced magnetic resonance imaging (MRI) using gadolinium, which demonstrated a soft amorphous tissue replacing the fat surrounding the left carotid bifurcation measuring 15 × 8 mm, with gadolinium enhancement, slight inflammatory hypersignal T1, absolute hypersignal T2, without any diffusion restriction ( Fig. 1 ). MRI showed no caliber differences nor atherosclerotic plaques regarding the carotid arteries (common, intern, and extern) on both sides.

Blood work revealed no anemia, a normal white blood cell count at 9 g/L, as well as a normal C-reactive protein level. Only a slight elevation of the erythrocyte sedimentation rate at 23 mm/h was noted. Antinuclear antibody test came back negative.

Based on the clinical and radiological examination and according to previous descriptions, the diagnosis of TIPIC syndrome was considered for our patient. A nonsteroidal anti-inflammatory drug (NSAID) regimen was initiated with ibuprofen for a total duration of 14 days; 600 mg three times a day for 7 days, then 400 mg three times a day.

**Fig. 2:**
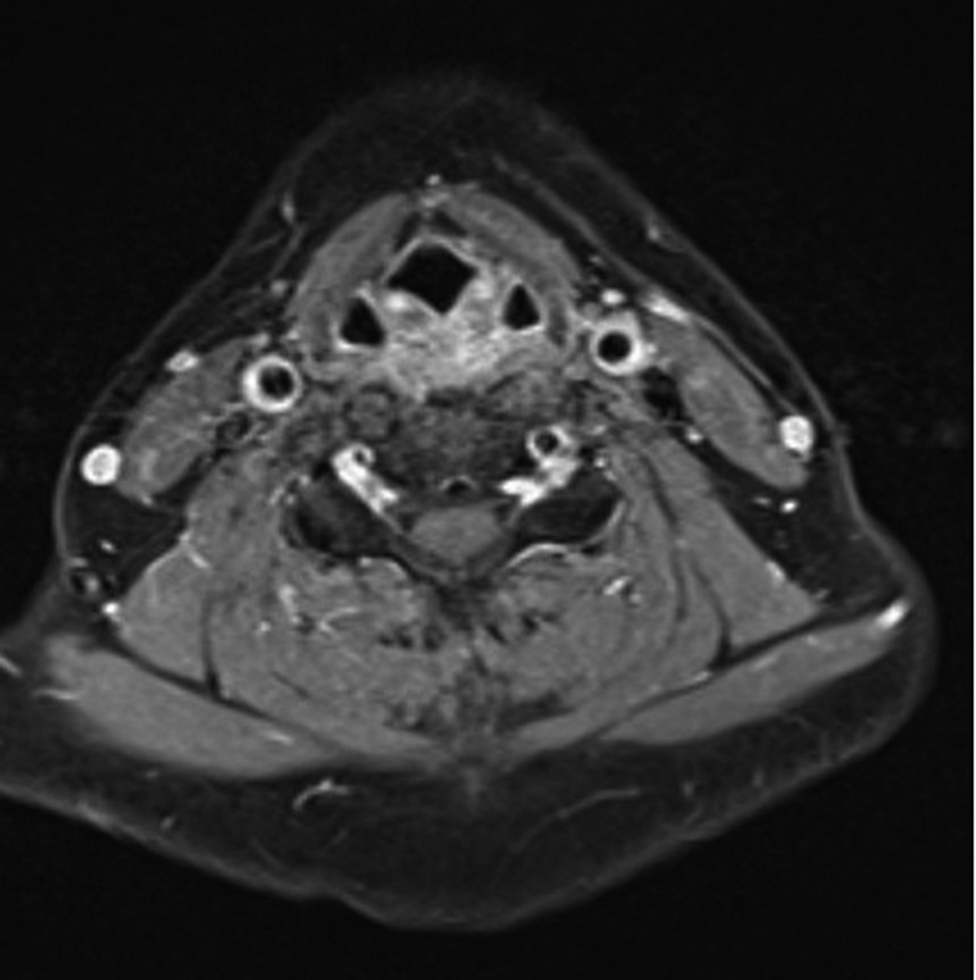
Magnetic resonance imaging at follow-up.

At the follow-up consultation 1 month after the treatment introduction, the patient reported a significant improvement of her symptoms. Nevertheless, a nocturnal pain was still present. We also performed a complete radiological follow-up, including ultrasound and MRI, that showed a complete resolution of initial findings particularly the absence of any PVI or amorphous tissue in the carotid bifurcation area ( Fig. 2 ).

## Discussion

TIPIC syndrome typically presents with unilateral or bilateral neck pain in the region of the carotid bifurcation that can be severe and persistent, often exacerbated by movement or pressure over the affected area. Its prevalence is estimated at 2.8% among subjects presenting with acute neck pain. 6 A slight female preponderance has been observed, with the highest incidence in the fifth and sixth decades of life, 2 but definite epidemiological data have been hard to pull out considering the small number of patient included in the available case series.

The exact cause of the syndrome remains unclear. Histological changes of TIPIC have been described for the first time by Upton et al, 7 with a predominantly lymphocytic proliferation with scattered neutrophils in the artery wall, with signs of fibrosis. Such findings suggested an inflammatory process, and the condition is considered to be idiopathic.

Diagnosis of TIPIC syndrome is based on clinical evaluation (presence of acute pain overlying the carotid artery, which may or may not radiate to the head) and exclusion of other conditions that can cause similar symptoms, such as infection (thyroiditis, sialadenitis), vascular disorders (dissection), other inflammatory conditions (vasculitis), or cervical mass. A thorough personal history and general patient examination including neurological and osteoarticular status should be routinely performed. Laboratory with inflammatory parameters (C-reactive protein, erythrocyte sedimentation rate [ESR], and white blood cells) is recommended. Inflammatory markers are usually not elevated in TIPIC. 8 9 Nevertheless, a discrete increase in ESR have been reported by some authors, 7 10 which correlates to our case.

Imaging plays a pivotal role in the diagnosis of TIPIC syndrome. MRI and ultrasound evaluation allows the exclusion of other pathological conditions of the artery and confirms the absence of structural disorders. Ultrasound evaluation typically shows an increased mural thickening associated with hyperechogenicity, corresponding to eccentric PVI. In a systematic, retrospective study, conducted by Lecler et al in 2017, PVI was most often in a posterior and lateral location on imaging studies. 6 The median largest axial diameter of the PVI ranged from 4 to 5mm. An intimal soft plaque was described in 58% ultrasound reviews and 27% of MRI reviews. No hemodynamic change was observed in color duplex Doppler. However, it should be noted that in this study, 4 of the 47 patients, didn't show PVI, which is a slightly atypical presentation. 6 MRI typically shows a thickened wall of the affected carotid artery, at the level of the carotid artery bifurcation. It is associated with increased contrast uptake, along with the presence of pericarotid infiltration and a T2 hyperintense captation. 11 The treatment of TIPIC syndrome is typically conservative and aimed at relieving symptoms. NSAID may be used to reduce inflammation and alleviate pain. Rest, local heat, and avoidance of pressure on the affected artery may also be recommended.

The prognosis of the syndrome is generally favorable, with most cases resolving spontaneously within a few weeks. In a recent multinational retrospective cohort study, the resolution of symptoms occurred after an average of 17 days, compared with 13 days as described in the literature. 12 Recurrence occurred in 20% of patients in the cohort study, but the condition is typically self-limiting and does not result in long-term complications or permanent damage to the carotid artery.

## Conclusions

TIPIC syndrome can be defined as follows: acute pain overlying the carotid artery, which may or may not radiate to the head; presence of eccentric PVI on imaging studies (ultrasound and/or MRI); exclusion of another vascular or nonvascular diagnosis; and improvement within 13 to 17 days either spontaneously or with NSAID treatment. We believe that multimodal imaging including Doppler ultrasound and MRI should be performed in the diagnosis process for every patient presenting unilateral or bilateral pain in the carotid bifurcation area. Those techniques would allow us to exclude any other pathology and confirm the diagnosis of this clinical–radiological entity.
